# Electronic Nose as a Novel Method for Diagnosing Cancer: A Systematic Review

**DOI:** 10.3390/bios10080084

**Published:** 2020-07-25

**Authors:** Chiara Baldini, Lucia Billeci, Francesco Sansone, Raffaele Conte, Claudio Domenici, Alessandro Tonacci

**Affiliations:** 1 School of Engineering, University of Pisa, Largo Lucio Lazzarino 1, 56122 Pisa, Italy; chiara.baldini16@gmail.com; 2 Institute of Clinical Physiology—National Research Council of Italy (IFC-CNR), Via Moruzzi 1, 56124 Pisa, Italy; lucia.billeci@ifc.cnr.it (L.B.); francesco.sansone@ifc.cnr.it (F.S.); raffaele.conte@ifc.cnr.it (R.C.); claudio.domenici@ifc.cnr.it (C.D.)

**Keywords:** artificial olfaction, cancer, electronic nose, health, sensors

## Abstract

Cancer is fast becoming the most important cause of death worldwide, its mortality being mostly caused by late or wrong diagnosis. Novel strategies have been developed to identify early signs of cancer in a minimally obtrusive way, including the Electronic Nose (E-Nose) technology, user-friendly, cost- and time-saving alternative to classical approaches. This systematic review, conducted under the PRISMA guidelines, identified 60 articles directly dealing with the E-Nose application in cancer research published up to 31 January 2020. Among these works, the vast majority reported successful E-Nose use for diagnosing Lung Cancer, showing promising results especially when employing the Aeonose tool, discriminating subjects with Lung Cancer from controls in more than 80% of individuals, in most studies. In order to tailor the main limitations of the proposed approach, including the application of the protocol to advanced stage of cancer, sample heterogeneity and massive confounders, future studies should be conducted on early stage patients, and on larger cohorts, as to better characterize the specific breathprint associated with the various subtypes of cancer. This would ultimately lead to a better and faster diagnosis and to earlier treatment, possibly reducing the burden associated to such conditions.

## 1. Introduction

Cancer is fast becoming the leading cause of death in higher income countries, with its mortality quickly reaching that of cardiovascular diseases [1]. Recent data from the US government estimated that more than 1.7 million new cases of cancer, with more than 600,000 deaths, could have occurred in 2018, with an estimated national expenditure for cancer care in the US reaching USD 147.3 billion in 2017 [2].

The magnitude of the problem has driven the scientists from all over the world to face the implementation of new strategies and tools for the detection, even at early stages, of various types of cancer from biological samples. This would ultimately facilitate early diagnosis, enabling the adoption of treatment strategies tailored at fighting the disease as fast as possible, thus increasing the possibility of success. One of such strategies is based on the development of the so-called “Electronic Nose” (E-Nose) systems, tools mimicking the functioning of the biological sense of smell whose first prototypes dated back to early 1980s [3]. Recently, such devices have been entering the universe of translational and clinical research as a useful alternative for analyzing gaseous samples to traditional, laboratory-based methodologies, including gas chromatography/ mass spectrometry (GC-MS), given their good portability, high customizability, quick response and relatively low cost [4,5].

Indeed, despite their effectiveness, sensitivity and, often, specificity, GC-MS technologies are time consuming, expensive and difficult to use in daily medical practice, as well as featuring low portability, making their employment feasible just in structured laboratory settings. Conversely, cheaper, portable E-Nose instruments are capable of detecting low concentrations of, and discriminating between, complex mixtures of volatile metabolites associated to several conditions without necessarily identifying individual chemical species [6,7].

Such negative sides of those more traditional methods paved the way to a broader development of E-Nose systems in several ways, including the study and design of new nanomaterials able to detect target chemical species (biomarkers) at very low concentrations in complex experimental settings (extremely high humidity, quick temperature variation, etc.). Within the framework of cancer research, E-Nose tools are commonly employed to look for a typical “breathprint”, capable of discriminating, at first level, patients suffering from cancer with respect to healthy controls and, if successful, to investigate the differences in the exhaled breath composition between categories of subjects with cancer based on the disease severity [8]. Finally, and most importantly from a clinical perspective, this approach could be used to diagnose cancer early, investigating breath composition unbalance at preliminary stages, paving the way for an unobtrusive alternative to expensive, poorly portable, invasive tools currently used for this aim [9].

Exhaled breath collection is normally performed by asking the individual to exhale within a hose, directly connected to a collection bag, usually made up of Tedlar. In order to minimize the influence of environmental compounds eventually present at the acquisition, the patient’s nose is often closed by means of a clip, and the portion of the exhaled breath to be analyzed is often represented by the sole measurement portion, previously separated by the dead space, being more important, as directly flowing from the lungs, thus containing all the compounds to be analyzed (see [10] for more details).

Once filtered, the sample is pushed within a measurement chamber by a pump, in order to interact with the sensors.

**Figure 1:**
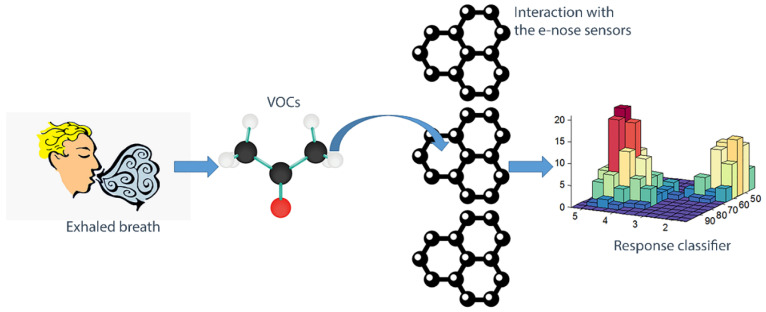
Simplified layout of the E-Nose principle of operation.

The measurement is performed on Volatile Organic Compounds (VOCs), being natural markers of pathophysiological mechanisms in the human body [11]. They are normally generated by biochemical processes, including oxidative stress and lipid metabolism, or can be absorbed from the external world through ingestion, inhalation or skin contact [12]. Once the VOCs are bounded to the detector, it is possible to obtain and analyze the associated electronic signal with specific software tools (Figure 1).

Finally, the breathprint of selected patients is compared with that from control subjects to compute sensitivity and specificity of the considered solution.

Taking into account the existing recent reviews dealing with the use of E-Nose in cancer diagnosis, the majority of them feature a deep focus on Lung Cancer (LC) [13,14,15] or are too widespread into medical diagnostics [16]. On the other hand, to the best of our knowledge, no recent reviews have summarized the use of E-Nose tools in the various types of cancer, therefore leaving a significant gap in the scientific literature.

Therefore, the aim of this systematic literature review is to provide an overall view on the state-of-the-art E-Nose systems to detect cancer from the exhaled breath of patients with various kinds of tumors, including, but not limited to, LC, head and neck squamous cell carcinoma (HNSCC), bladder cancer (BlC), colon or colorectal cancer (CRC). In addition, given the literature’s recently published findings, and taking into account the quick development of Information and Communication Technologies, we also tried to hypothesize some future development possibly making the E-Nose approach at the clinical forefront in next years.

After this introductory section, Section 2 will focus on the methods used for this literature search, Section 3 will briefly present the main results obtained, and will discuss the results based on the different types of tumors actually studied. Finally, Section 4 briefly outlines some conclusions and future developments, with practical messages addressed to the reader.

## 2. Materials and Methods

A systematic literature review was conducted, following the PRISMA guidelines [17], on PubMed database, according to the following terms: ((“E-nose” OR “Electronic nose” OR “Olfaction technology” OR “Odor detection”) AND (“Cancer” OR “Carcinoma” OR “Adenocarcinoma” OR “Tumor” OR “Malignancy” OR “Malignant disease”)).

The records included were related to articles published up to 31 January 2020 in English language, excluding systematic reviews, meta-analysis and case report studies.

Each article was analyzed in terms of disease investigated, technology employed, sensitivity and specificity or accuracy.

## 3. Results

**Figure 2:**
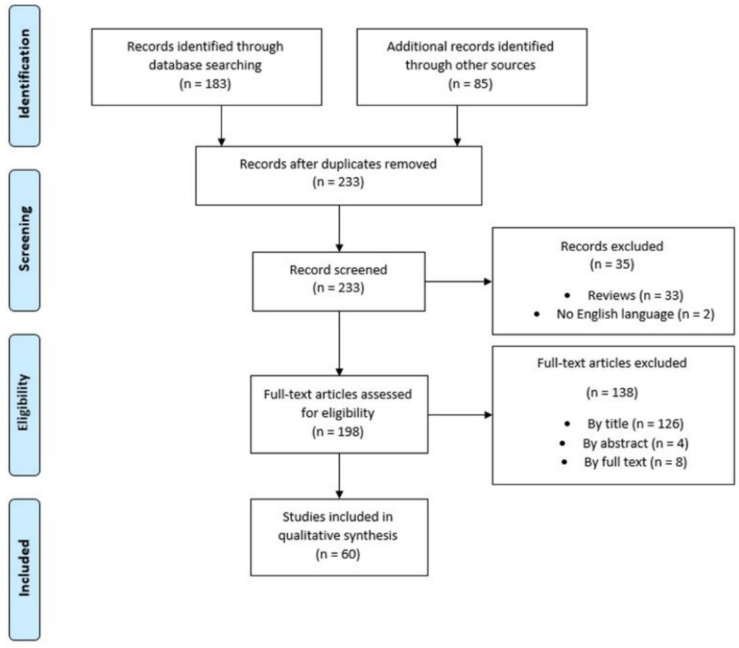
PRISMA flowchart related to the literature review.

Figure 2 displays the flowchart related to the literature review.

Of the 268 articles initially retrieved, based on the inclusion and exclusion criteria applied, 60 records have been retained for inclusion in the qualitative synthesis foreseen by the systematic review. The majority of such works dealt with lung cancer, the recognized major cause of death for cancer around the world for both men and women [18]. Other records included studies dealing with HNSCC, CRC, OC, prostate cancer (PC), gastric cancer (GC), bladder cancer (BlC), malignant melanoma (MM), breast cancer (BrC), kidney cancer (KC).

The included articles are displayed in Table 1.

### 3.1. Lung Cancer

**Figure 3:**
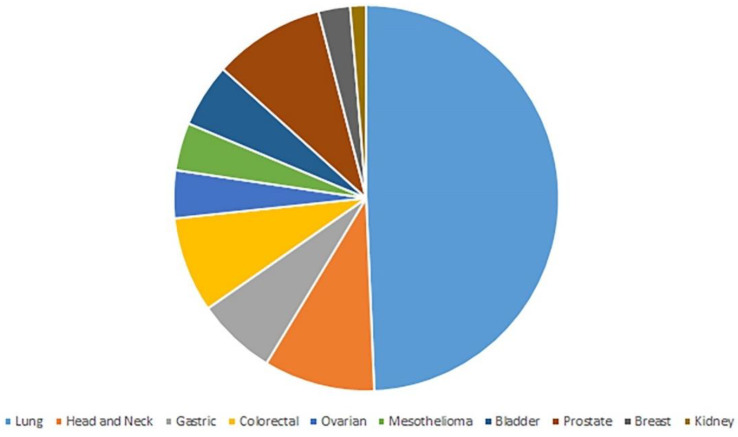
Distribution of cancer types across the retrieved studies.

As reported in the table, and visually displayed in Figure 3, the majority of the studies retrieved in the present literature review concern LC, appearing to be the main cause of death from cancer worldwide [18]. Overall, the literature confirms the good detection ability of the majority of E-Nose systems for LC, although still displaying wide heterogeneity mainly due to: (i) acquisition protocol, (ii) tool employed and, (iii) sample size, open issues for the majority of studies dealing with E-Nose in all cancer types.

Regarding technologies, out of 37 articles about LC, 13 have used nanosensors, both embedded into commercial devices, like the popular Cyranose 320 (Sensigent, Baldwin Park, CA, USA), and put as a non-commercial products and prototypes. Indeed, nanosensors offer many advantages over other technologies, including a fast response time, reasonable detection limits, high portability and scalability, good sensitivity and resolution, eventually high selectivity [78]. In the majority of the studies employing such technology, results in terms of sensitivity, specificity and accuracy were satisfying (mostly above 80% of correct accuracy in discriminating between LC and controls and between LC and other cancer types), still considering the low specificity of E-Nose systems in general.

Among the first articles published with this approach on this specific population was the one by Machado et al. in 2005 [21], discriminating LC and controls by mean of Cyranose 320 with a 71.4% of sensitivity and 91.9% of specificity. The Cyranose 320 is a portable chemical vapor detector, consisting of 32 composite carbon black polymer sensors. VOCs in exhaled air bind to the polymers, generating a reversible change in the electrical resistance of the sensors. Together, all sensor deflections form a specific ‘breathprint’. Here, discrimination analysis was performed using a prior filter, followed by Principal Component Analysis (PCA) to reduce data complexity, classes’ separation computation via Mahalanobis distance and, finally, Support Vector Machine (SVM) analysis for classification. VOCs investigated included Isobutane, Methanol, Ethanol, Acetone, Pentane, Isoprene, Isopropanol, Dimethylsulfide, Carbon disulfide, Benzene and Toluene, representing a quite complex mixture of volatiles eventually present in the exhaled breath of diseased individuals. Some of those compounds were also studied by Di Natale et al. [19], using aniline, alkanes, and benzene derivatives, by D’Amico et al. and Tran et al. [26,28], using aniline, o-toluidine and cyclopentane, and by Peng and colleagues [27], in turn employing isoprene, alkanes, methylalkanes and benzene derivatives as disease biomarkers.

However, with the technological scaling-up taking place, the results obtained with nanosensors also progressively improved. Indeed, just a few drawbacks were noticed throughout the years, with poor performances and tricky airflow control in some specific tasks observed in the study by Bikov et al. [10], evaluating VOCs applying PCA for discriminating between classes, where just 40% of specificity in discriminating between LC and healthy smokers was obtained, or in the article by Hubers et al. [37], that failed to retrieve good results in terms of specificity to discriminate between patients with LC and controls with COPD. The features were reduced by means of a PCA with 6 components, one of which (PC4) performed statistically better in discriminating categories, therefore it was used to construct a Receiver Operating Characteristic (ROC) curve to calculate sensitivity and specificity.

Thanks to the feedbacks obtained in such trials, and taking advantage of new technological advances, Sensigent developers have obtained continuously better performances by their product, as demonstrated by the large-scale studies published by Tirzīte et al. in 2017 and 2019 [49,54]. Here, the Cyranose 320 showed its ability to discriminate between LC and controls as well as between different stages of LC, using Support Vector Machine (SVM) and Logistic Regression Analysis (LRA), respectively, with a good performance in terms of accuracy.

The improvement of Cyranose 320 performances foreseen in the near future should mainly regard the optimization of specificity, reducing the false positive still noticed. In addition, as observed by Huang and colleagues [50], that used Linear Discrimination Analysis (LDA) and SVM for classification, temperature and humidity should be considered carefully and maintained fixed at least within the testing room.

Another frequently used technology in the E-Nose applications specifically designed for LC discrimination is represented by MOS sensors, still being, after many years, among the leading technologies in the specific field. MOS ensure a wide range of response, high sensitivity, low cost, fast response time, small size, easy fabrication, long-lasting life and wide temperature operating range. Nevertheless, they appear to be highly selective, quite power expensive, poorly stable, and highly sensitive to environmental conditions, making the control of such variable particularly critical [79].

Within the cancer research, this approach was first used in 2007 by Blatt and colleagues [22], using non-parametric LDA and several supervised pattern classification techniques, with satisfying results in terms of correct classification between LC and controls on a quite large population, accounting for N = 101 subjects divided into the two groups, correctly classified in more than 90% of cases. More recently, large studies were published using commercial devices relying on this driving principle, including the Aeonose (The eNose Company, Zutphen, The Netherlands), employed by most Dutch studies published (e.g., those by van Hooren, Kort and van de Goor [44,51,52]), discriminating between LC and controls, as well as between LC and other non-cancer disturbances, based on VOCs, in more than 80% of cases. More specifically, the Aeonose consists of three micro hotplate metal oxide sensors. During the measurement, the hotplates are periodically heated and cooled between 260 and 340 °C in 32 steps, during which they are exposed to the exhaled breath. The redox reactions of the VOCs at the surface of the MOS result in a change in conductivity of the sensors, creating a unique pattern of redox reactions of the VOCs.

Van Hooren and colleagues discriminated between LC and controls after a processing for data compression and Artificial Neural Networks (ANN), followed by Matthews Correlation Coefficients (MCC) to measure the quality of binary classifications. A best-fit model of the data was then calculated. Finally, to predict the fit of a model for future undefined breath samples, cross-validation of the data was performed using leave-one-out.

A very similar approach was applied by Kort et al. [51] and van de Goor and colleagues [52], using the ad-hoc software for data processing Aethena (The eNose Company, Zutphen, The Netherlands), performing pre-processing of data, data compression, leave-10%-out cross-validation, model selection, and combining prediction models with promising AUCs.

As in the case of the previously described Cyranose 320, some issues have been noticed, with Aeonose failing to reach a satisfying result in terms of specificity in early discriminating LC and controls, but representing, to date, the only evidence of MOS sensors failure in the specific field of LC.

Quartz microbalance (QMB) technologies were also used in a few articles dealing with LC, mostly by Italian groups, displaying satisfying results.

A handful studies also employed other, mixed approaches.

Shlomi et al. [48] have applied a unique set of 40 sensors, particularly chemiresistors, based on two types of nano-materials (i.e., GNPs, Graphene Nanoplatelets and SWCNTs, Single-Wall Carbon Nanotubes). The sensors used were either based on (i) organically stabilized spherical GNPs (core diameter 3–4 nm), or (ii) SWCNTs capped with polycyclic aromatic hydrocarbon (PAH) or hexabenzocoronene (HBC). Such sensors typically feature a fast, reversible response when exposed to typical breath VOCs.

One hundred and nineteen individuals were enrolled for the study, 16 of which with Early Lung Cancer (ELC), 73 with Advanced Lung Cancer (ALC) and 30 with benign nodules. ELC patients were identified with respect to individuals with benign nodules with a sensitivity of 75% and a specificity of 93.3% using Discriminant Function Analysis. The main limitations of the approach proposed reside in the relatively low sample size, in the consideration of benign nodules after 2 years of stable X-rays analysis and in possible “recall biases” to which the smoking history condition can be subjected.

Li et al. [46] investigated the usefulness of E-Nose systems in the field of LC using a 14-sensor array, of which eight were MOS, four electrochemical sensors and two temperature/humidity detectors. The gas reaction chamber, made of aluminum alloy, with a volume of about 220 mL, hosted sensor probes. When the gas to be tested passes through the gas chamber, the sensor array responds in terms of voltage (or current) signals. After initial processing (filtering, amplification, etc.), signals are sent to the main control chip (STM32F10) for analog-to-digital conversion and temperature/humidity compensation, and finally to the computer through USB for display and storage. Twenty-four patients with LC, 5 subjects with respiratory conditions, 10 healthy smokers and 13 healthy non-smokers were enrolled. After elimination of the 5 patients with respiratory conditions, the 47 remaining subjects were divided into LC and controls, and the discrimination analysis between those two classes provided a 91.58% of sensitivity and 91.72% of specificity. This analysis was performed, after an initial data pre-processing, by using different dimension reduction methods, including PCA, LDA, Laplacian Eigenmap (LE), local linear embedding (LLE), and t-Stochastic Neighbor Embedding (tSNE). Finally, classification was done with fuzzy k-NN and SVM.

Overall, research on LC using E-Nose systems, populated by a growing number of studies, displayed satisfying results even with different technologies, making it a field where such approach can be used more largely even in clinical practice, with slight technological development eventually desirable.

### 3.2. Head and Neck Carcinoma

LC was the most frequently analyzed type of cancer, although different tumors are taken into account in studies employing the E-Nose, as well.

Among them, HNC was studied in seven articles retrieved in this review, some of which not specifically dealing with HNC but taking into account this kind of tumor together with other ones.

Here, probably due to their wider spread within the scientific community and to their low cost, MOS sensors are mostly used, with four out of seven articles following this approach, especially using the commercial Aeonose. Good sensitivity, specificity and accuracy are displayed, with values somewhat similar to what is obtained in LC.

For example, among them, van Hooren et al. [44] used E-Nose to characterize the exhaled breath of 52 patients with HNC and 32 individuals with LC, distinguished with 85% sensitivity and 84% specificity, although wider groups are desirable to allow conducting analyses on subgroups based on the disease severity.

Similarly, van de Goor and colleagues [57] used 5 Aeonose tools to distinguish between various types of cancer (N = 100 HNC, N = 28 colon cancer, N = 40 bladder cancer). Data analysis was performed with the proprietary software package Aethena with results presented in a scatter plot and a ROC curve. Matthews correlation coefficients were calculated to determine the quality of the binary classifications and 95% confidence intervals were given. This approach revealed a 79% sensitivity and 81% specificity between HNC and colon cancer, an 80% sensitivity and 86% specificity between HNC and bladder cancer, and an 88% sensitivity and 79% specificity between bladder cancer and colon cancer. The main confounding factor is that most patients with HNSCC were heavy smokers with advanced stage tumors, whereas patients with bladder or colon cancer were not diagnosed to exclude HNC.

Nanosensors were used in two out of seven papers, both published by Israeli groups and displaying excellent performances of the NA-NOSE device. Hakim and colleagues [29] collected exhaled alveolar breath and analyzed it with an array containing five sensors based on spherical gold nanoparticles (GNPs) with tert-dodecanethiol, hexanethiol, 2-mercaptobenzoazole, 1-butanethiol, and 3-methyl-1-butanethiol ligands. The NA-NOSE data were analyzed by PCA and, in a complementary approach, SVM was used to classify the principal component data and cross-validation was applied to evaluate the sensitivity and specificity obtained. With such approach, the authors obtained 100% sensitivity in discriminating HNC and controls (with 92% specificity) and 100% of both sensitivity and specificity when trying to differentiate HNC from LC.

Later on, Gruber et al. [55], on a larger cohort made up of 68 patients with HNC and 19 healthy controls, distinguished, via DFA, between HNC and controls with 77% sensitivity and 90% specificity and between different sites of the tumor (larynx vs. pharynx) with 90% accuracy. Finally, early and late stage tumors were stratified with 95% accuracy, fostering the application of this tool in the specific field.

As for LC, also for HNC results obtained are satisfying, suggesting a step forward towards the use of E-Nose technology even in clinical practice.

### 3.3. Prostate Cancer

Prostate cancer was also investigated in seven articles retrieved in the present review. Among them, both MOS sensors and QMB-based tools were applied twice, with satisfying results, somewhat comparable to those obtained in LC and HNC. Concerning the biomarkers studied, 2,6-dimethyl-7-octen-2-ol, pentanal, 3- octanone and 2-octanone were mostly used [26,73,74], but Peng and colleagues also used Toluene, 2,3,4-trimethyl decane, pxylene and 2,2-dimethyl decane [27]. In order to get more reliable results, the investigation of wider cohorts is needed, aiming at understanding whether this approach could be translated into the clinics.

### 3.4. Colorectal Cancer

Colorectal adenocarcinoma was studied in six articles, three of which were conducted with nanosensors. In terms of sample size, the study by Amal and colleagues [65] resulted to be the largest, performed on 65 patients with CRC, 22 with adenomas and 122 control subjects. Breath samples were analyzed through a system composed of six sensors either based on (i) organically stabilized spherical GNPs, with a 3–4-nm core diameter, and (ii) SWCNTs. Four different organic functionalities of the GNP sensors and two for the SWCNT contributed to the chemical diversity of the sensors. The organic ligands of the GNPs provided broad cross-reactive absorption sites for the common breath VOCs. In the study, the accuracy of discrimination was 91% between CRC and controls, with 85% sensitivity and 94% specificity.

Satisfying results were also obtained by Westenbrink et al. [64] using WOLF eNose (composed of 13 sensors, of which eight amperometric electro-chemical sensors, two non-dispersive infrared optical devices and a single photo-ionization detector) for discriminating levels of 2-methyl-3-phenyl-2-propenal, pcymene and anisole, and by de Meji and colleagues [63], taking advantage of the good performances of the Cyranose 320 in discriminating CRC and controls based on VOCs profile (85% and 87% of sensitivity and specificity, respectively). The relatively low number of studies published to date suggests that the use of E-Nose technologies in the clinical practice appears still premature; however, if confirmed on further studies, possibly on larger cohorts, such findings could pave the way for the preliminary adoption of such approach within screening studies and to control disease progression.

### 3.5. Gastric Carcinoma

The use of E-Nose technology is particularly important in those conditions where early detection is crucial to decrease the cancer mortality. Among them, gastric carcinoma is considered among the main fields of application, being the fourth most common type of tumor [80] and featuring high mortality due to its late identification [81].

In terms of E-Nose tools, GC was commonly studied with nanosensors, appearing in three out of the five studies published to date, using both self-made and commercial devices. Among the latter, Schuermans et al. [62] applied the E-Nose technology to this field, specifically using the Aeonose to classify breath samples from 16 patients with GC and 28 controls. The ROC curve derived from the analysis and obtained with the Aethena tool, revealed 81% sensitivity and 71% specificity for the abovementioned discrimination, highlighting as main limitations the small sample size and the specificities of the Chinese population on which the investigation has been carried out.

Given the importance of early detection in general within oncology, but in particular as concerns GC, future studies dealing with E-Nose systems should be more focused on the early identification of this kind of tumors, evaluating their efficacy and potential transferability into the practice.

### 3.6. Other Conditions

A handful studies also dealt with the use of E-Nose technology in other cancer conditions. They include Bladder Cancer (BlC), displaying four studies using QCM, nanosensors, MOS and carbon nanotubes; Ovarian Cancer (OC), investigated in three studies with heterogeneity in terms of the method employed; Malignant Melanoma (MM), where the Cyranose 320 was the sole device applied; Breast Cancer (BrC), where nanosensors were applied; and Kidney Cancer (KC), with the use of carbon nanotubes.

All those works reported satisfying results, except for some specific comparisons performed by Lamote et al. [70], where Cyranose 320 was far from being optimal in discriminating MM and controls, probably due to the small sample size, and for some clinical characteristics of OC deeply investigated by Amal and colleagues [66] where the NA-NOSE needed some improvement in discriminating, for example, OC stages, possibly due to the complexity of the task demanded.

## 4. Major Issues

E-Nose systems also account for a number of issues, mostly depending on the nature of the detectors constituting the sensing part of the device.

At first, such systems report troubles when asked to discriminate and quantify odors in very small concentrations. This fact can be related either to technological constraints, with limits of detection not reached by eventually low concentrations of the target molecule, or to customization issues, with a strong need for tailoring the selection of sensors to be employed for the array constituting the E-Nose that is not always optimally matched. In both cases, such limits bring to low-amplitude or noisy signals, which might be confounded from other signals with similar amplitudes, increasing the need for an improvement in sensor fabrication and pattern recognition algorithm definition, topics of great interest in this specific field in recent years [82].

Second, the response characteristics of sensors composing the E-Nose systems should be carefully taken into account, including the significant sensor drift, especially when exposed repeatedly to gas mixtures within short time frames, or in the presence of extremely high humidity conditions or sudden temperature variations. Such issues could lead to false diagnostics, significantly decreasing the reliability of E-Nose-produced data. Therefore, a careful control of airflow, as well as of other environmental variables including temperature and humidity, is mandatory and is commonly employed in current research protocols in order to achieve reliable data, cleaned from those, non-desirable external factors.

Additionally, the employment of single-use discardable sensors could be a solution, somewhat helping to promote standardization for volatilome databases to be created [83]. Otherwise, every single system would need to have its exclusive training algorithm, and renewed constantly due to sensor decay, making this technology too demanding to implement in clinical practice.

Another issue of such tools includes the need for realizing E-Nose devices with wireless capabilities, in order for them to be included within large wireless sensor nodes. This would allow such devices to be provided by hospitals and healthcare providers to the patients for their self-monitoring at home, within the framework of patient empowerment and p4 medicine, granting to the medical doctors a continuous control to the health status of a patient and allowing, at the same time, the achievement of an uniform measurement protocol, overtaking one of the major issues actually present with E-Nose devices [15]. This step is characterized by the need for controlling total power consumption, complexity, costs and data acquisition rate, current issues actually in the process to be solved by manufacturers and developers [84,85].

Finally, a further key-point for the good success of E-Nose systems is represented by their wearability, allowing them to be used for real time monitoring of breath and sweat while embedded into the textiles. Despite being hard to achieve, this characteristics has been largely taken into account in recent times, making the integration within garments easier than before, driving E-Nose tools to be part of complete health care systems, especially for several groups of patients and athletes in general [86].

## 5. Conclusions and Future Developments

In light of the evidence in the literature, breath analysis represents a promising alternative to traditional methods within the framework of cancer diagnosis, being fast, unobtrusive and practical.

Taking into account the results of the studies retrieved in the present investigation, promising data were observed when distinguishing patients with LC and controls. The main confounders in this analysis were related to the device used, the population studied concerning age and ethnicity, the sample size, which accounts for being one of the major limitations of the studies retrieved, making the management of data mining quite tricky, the statistical methods used and eventual habits, including smoking. In fact, the latter factor is known to significantly alter the VOC composition, as happens with diet and drugs use.

Concerning the E-Nose technology, the most widely used tools are based on nanosensors, QMB and MOS sensors, overall displaying good performances in discriminating cancer patients and controls. Such data are particularly true when investigating large-scale cancer groups, including LC, HNC or PC, and should be further confirmed on less prevalent tumors. In addition, other technologies can flank such tools with the aim of improving E-Nose discriminatory capabilities.

The main limitation of the different studies published to date is that some of them take into account patients with late stages of cancer, whereas the early diagnosis is not always investigated, and often not exhaustively. This should be carefully overcome in future research to further highlight the pivotal contribution eventually borne by the E-Nose approach.

Finally, more studies are needed, especially on larger cohorts, to better investigate the most suitable E-Nose tools for identifying the various types of cancer, in order to standardize breath analysis with this method and to avoid confounders that could negatively affect the integrity of data. Such a point should be solved, since the E-Nose technology exists since many decades ago and it is widely employed within this field since 2003 at least; however, despite the promising results reported in the scientific literature, and mentioned in the present work, its use in clinical practice is still extremely limited. The development of small, wireless E-Nose tools, easy to use, economically viable and with little power consumption, possibly interfaced to smartphones, could allow patients to be self-monitored and continuously tracked at their home, detecting unbalance related to the VOC composition that might possibly trigger an alarm for doctors and caregivers, suggesting the need for more frequent visits and medical check-ups. This would enable large-scale screening on the overall population eventually at risk for cancer due to particular conditions (i.e., when exposed to pollutants or radiations, when displaying risky behaviors like being heavy smokers, when having familiarity for cancer, etc. [87,88,89,90]) and more precise control of the disease progression in patients with early stages of cancer.

In this overall framework, the solution proposed, probably solving some of the issues still present, would enable the large-scale employment of the E-Nose technology and the delivery of fast, reliable and economically viable results of some clinical analysis involving breathomics. Indeed, optimized solutions, based on such technology, could bring to a better, earlier cancer diagnosis, decreasing at the same time the misdiagnosis and the mortality associated with cancer.

## Figures and Tables

**Table 1:** Studies included in the literature review (AC: adenocarcinoma, AEx: asbestos-exposed, ALC: advanced lung cancer, ARD: asbestos-related diseases, BC: bronchogenic carcinoma, BlC: bladder cancer, BrC: breast cancer, CC: colon cancer, COPD: chronic obstructive pulmonary disease, CRC: colorectal cancer, ELC: early lung cancer, GC: gastric carcinoma, GC-MS: gas chromatography–mass spectrometry, GNP: gold nanoparticles, HNC: head and neck squamous cell carcinoma, IBS: irritable bowel syndrome, KC: kidney cancer, LC: lung cancer, MM: malignant mesothelioma, MOS: Metal Oxide Semiconductor, N/A: (quantitative) data not available, NSCLC: non-small cell lung cancer, OC: ovarian cancer, PC: prostate cancer, QMB: quartz microbalance, SAW: Surface Acoustic Wave, SCC: squamous cell carcinoma, SCLC: small cell lung cancer, TCC: transitional cell carcinoma) (* data depending on the validation model used).

Study	Participants	Cancer Type	Groups	Aim	Device	Sensitivity (%)	Specificity (%)	Accuracy (%)
[19]	N = 60	LC	LC (n = 42), controls (n = 18)	LC (a) and controls (b) classification	LibraNose			100 (a) 94 (b)
[20]	N = 50	LC	LC (n = 24), other non-cancer chronic lung conditions (n = 8), healthy controls (n = 18)	Discrimination between LC and controls	SAW-based eNose	N/A	N/A	N/A
[21]	N = 135	BC	BC (n = 28), healthy controls (n = 107)	To discriminate between cancer and controls	Cyranose 320	71.4	91.9	
[22]	N = 101	LC	LC (n = 43), controls (n = 58)	Correct classification between LC and controls	MOS sensors-based eNose	95.3	90.5	92.6
[23]	N = 143	NSCLC	NSCLC (n = 49), COPD (n = 18), idiopathic pulmonary fibrosis (n = 15), pulmonary artery hypertension (n = 20), sarcoidosis (n = 20), healthy controls (n = 21)	Prediction of lung cancer	Colorimetric sensor array	73.3	72.4	
[24]	N = 32	LC	LC (n = 15), other non-cancer chronic lung conditions (n = 7), healthy controls (n = 10)	Discrimination between LC and controls	SAW-based eNose	N/A	N/A	N/A
[25]	N = 30	NSCLC	NSCLC (n = 10), COPD (n = 10), healthy controls (n = 10)	Discrimination between: NSCLC and COPD (a) and between NSCLC and controls (b)	Cyranose 320			85 (a) 90 (b)
[26]	N = 92	LC	LC (n = 28), other lung diseases (n = 28), controls (n = 36)	To discriminate between LC and controls (a) and between LC and other lung diseases (b)	LibraNose	85 (a) 93 (b)	100 (a) 79 (b)	
[27]	N = 177	LC, CC, BrC, PC	LC (n = 30), CC (n = 26), BrC (n = 22), PC (n = 18), healthy controls (n = 81)	Discrimination between cancer and controls, and between the different types of cancer	Nanosensors based on organically functionalized GNP coupled with GC-MS	N/A	N/A	N/A
[28]	N = 89	LC	LC (n = 16), other respiratory disorders (n = 11), smokers (n = 18), ex-smokers (n = 11), non-smokers (n = 33)	Discrimination between LC and controls	ENS Mk 3			p-values of 0.045, 0.025 and 0.001 for discrimination according to the different eNose channels
[29]	N = 87	LC, HNC	LC (n = 25), HNC (n = 22), healthy controls (n = 40)	Discrimination between: LC and controls (a), HNC and controls (b), LC and HNC (c)	Nanoscale NA-NOSE	100 (a, b, c)	92 (a, b) 100 (c)	
[30]	N = 18	LC	LC (n = 9), controls (n = 9)	Discrimination between cases and controls	Semiconductor and electrochemical-based CN e-Nose II	100	88.9	94.4
[31]	N = 229	LC	LC (n = 92), controls (n = 137)	Discrimination between cases and controls	Colorimetric sensor array			81.1
[32]	N = 72	LC	LC (n = 53), controls (n = 19)	Discrimination between: LC and controls (a), adeno and squamous carcinoma (b) and between early and advanced stage of disease (c)	Nanoscale NA-NOSE, coupled with GC-MS			88 (a, b, c)
[33]	N = 30	LC	LC (n = 20), of which AC (n = 10) and SCC (n = 10); healthy controls (n = 10)	Discrimination between cases and controls (a), and between cancer subtypes (b)	QMB-based eNose	97.5 (a)	75 (a)	90 (a) 75 (b)
[34]	N = 89	LC	LC (n = 47), healthy controls (n = 42)	Discrimination between cases and controls	MOS-SAW-based eNose	93.62	83.37	
[35]	N = 17	LC	LC (n = 12), healthy controls (n = 5)	Discrimination between cases and controls	Nanomaterial-based eNose	100	80	
[10]	N = 64	LC	LC (n = 27), healthy controls (n = 37)	To discriminate between LC and controls (a), LC and healthy smokers (b), LC and healthy never-smokers (c). Then, LC and controls were compared at higher expiratory rate (d), and after 10s of breath hold (e)	Cyranose 320	63 (a) 96 (b) 67 (c) 81 (d) 78 (e)	78 (a) 40 (b) 81 (c) 76 (d) 65 (e)	72 (a) 81 (b) 74 (c) 78 (d) 70 (e)
[36]	N = 30	LC	LC (n = 20), other lung diseases (n = 10)	Discrimination between LC and controls	QMB-based eNose coupled with GC-MS			90
[37]	N = 77	LC	LC (n = 38), COPD controls (n = 39)	To discriminate between LC and controls	Cyranose 320	80	48	
[38]	N = 144	LC	LC (n = 31), COPD (n = 31), asthma (n = 37), controls (n = 45)	Discrimination between: COPD vs. LC (a), asthma vs. LC (b), controls vs. LC (c)	SpiroNose			87(a), 68 (b), 88 (c)
[39]	N = 191	LC	LC (n = 25), current or former heavy smokers without LC (n = 166)	To discriminate between LC and controls	Cyranose 320	88	81.3	
[40]	N = 100	LC	LC (n = 23), controls (n = 77)	Discrimination between LC vs. controls	BIONOTE	86	95	
[41]	N = 146	LC	LC (n = 70), controls (n = 76)	Discrimination between LC vs. controls	LibraNose	81	91	
[42]	N = 39	LC	LC (n = 39)	Discrimination based on disease progression: disease control vs. baseline (a), disease control vs. progressive disease (b)	Nanoscale NA-NOSE, coupled with GC-MS	93 (a) 28 (b)	85 (a) 100 (b)	89 (a) 92 (b)
[43]	N = 37	LC	Treatment naïve LC (n = 12), former or current smokers with COPD (n = 12), healthy never-smoking controls (n = 13)	To discriminate between LC and controls (a), to identify LC in current and former smokers (b), to identify LC in non-smokers (c)	Chemiresistor-based alkane eNose	83 (a) 86 (b) 80 (c)	88 (a) 80 (b) 93 (c)	
[44]	N = 84	HNC and LC	HNC (n = 52), LC (n = 32)	Discrimination between HNC vs. LC	Aeonose	85	84	
[45]	N = 129	LC	LC (n = 57), controls (n = 72)	Discrimination between LC vs. controls	zNOSE4200	76	94	
[46]	N = 52	LC	LC (n = 24), other respiratory conditions (n = 5), healthy smokers (n = 10), healthy non-smokers (n = 13)	Discrimination between LC vs. healthy subjects	Different sensors	91.58	91.72	
[47]	N = 813	LC, CRC, HNC, OC, BlC, PC, KC, GC, other non-cancer conditions	LC (n = 45), CRC (n = 71), HNC (n = 22), OC (n = 48), BlC (n = 73), PC (n = 11), KC (n = 33), GC (n = 99), other non-cancer conditions (n = 411)	Correct discrimination between the conditions	Au nanoparticles and single-walled carbon nanotubes			86 (average accuracy of all classifiers)
[48]	N = 119	LC	ELC (n = 16), ALC (n = 73), benign nodules (n = 30)	Discrimination between ELC vs. benign nodules	Nano-materials-based sensor array	75	93.3	
[49]	N = 335	LC	LC (n = 165), non-cancer (n = 91), controls (n = 79)	To discriminate between: cancer and non-cancer (a); cancer and healthy controls (b); lung cancer stages (c)	Cyranose 320	87.3 (a)	71.2 (a)	93 (b) 78.5 (c)
[50]	N = 244	LC	LC (n = 56), controls (n = 188)	LC detection	Carbon nanotubes sensor array	From 75 to 100 *	From 86.2 to 96.6 *	From 85.4 to 92.7 *
[51]	N = 290	LC	LC (n = 144), healthy controls (n = 146)	Prospective, multi-centre study, to: early discriminate patients with NSCLC and healthy subjects (a); discriminate between subtypes of NSCLC, notably: AC (b), SCC (c), NSCLC (d)	Aeonose	94.4 (a) 81.5 (b) 80.8 (c) 88.9 (d)	32.9 (a)	
[52]	N = 145	LC	LC (n = 52), benign disturbances (n = 93)	Discrimination between LC vs. benign disturbances	Aeonose	83	84	
[53]	N = 16	LC	LC (n = 6), controls (n = 10)	Classification of the two classes of subjects: LC (a), controls (b), overall (c)	MOS sensor array	85.7 (a)	100 (b)	93.8 (c)
[54]	N = 475	LC	LC (n = 252), non-cancer controls (n = 223)	Discrimination between LC and controls among smokers (a) and non-smokers (b)	Cyranose 320	95.8 (a) 96.2 (b)	92.3 (a) 90.6 (b)	
[55]	N = 87	HNC	HNC (n = 68), healthy controls (n = 19)	Discrimination between: HNC and controls (a), HNC and benign tumor (b), HNC at larynx and pharynx (c), early and late stage (d)	Nanoscale NA-NOSE, coupled with GC-MS	77 (a, b)	90 (a, b)	83 (a, b) 90 (c) 95 (d)
[56]	N = 59	HNC	HNC (n = 36), controls (n = 23)	Discrimination between cases and controls	MOS-based eNose	90	80	
[57]	N = 168	HNC, BlC and colon cancer	HNC (n = 100), bladder (n = 40), colon (n = 28)	Discrimination between: HNC vs. colon cancer (a), HNC vs. bladder cancer (b), colon vs. bladder cancer (c)	Aeonose	79 (a), 80 (b), 88 (c)	81 (a), 86 (b), 79 (c)	
[58]	N = 40	HNC	Recurrent HNC (n = 20), not recurrent HNC (n = 20)	Discrimination between recurrent and non-recurrent HNC	Aeonose	85	80	83
[59]	N = 130	GC	GC (n = 37), ulcers (n = 32), less severe conditions (n = 61)	Discrimination between: GC and benign conditions (a), early and late stage GC (b), ulcers and less severe conditions (c)	Nanoscale NA-NOSE, coupled with GC-MS	89 (a, b) 84 (c)	90 (a) 94 (b) 87 (c)	
[60]	N = 103	GC	GC (n = 30), controls (n = 73)	Discrimination between cases and controls (a) and between different cancer levels (b)	Silicon nanowire field effect transistor	71 (a) 92 (b)	89 (a) 67 (b)	85 (a)
[61]	N = 484	GC	GC (n = 99), other conditions (n = 385)	Discrimination between GC and controls	Nanoscale NA-NOSE, coupled with GC-MS	73	98	92
[62]	N = 44	GC	GC (n = 16), controls (n = 28)	Discrimination between GC vs. controls	Aeonose	81	71	
[63]	N = 157	CRC	CRC (n = 40), advanced adenoma (n = 60), healthy controls (n = 57)	Discrimination between: CRC and controls (a), advanced adenoma and controls (b)	Cyranose 320	85 (a) 62 (b)	87 (a) 86 (b)	
[64]	N = 92	CRC	CRC (n = 39), IBS (n = 35), controls (n = 18)	CRC vs. IBS identification	WOLF	78	79	
[65]	N = 209	CRC	CRC (n = 65), adenomas (n = 22), controls (n = 122)	Discrimination between: CRC and controls (a), advanced and non advanced adenoma (b), advanced adenoma and controls (c)	Cross-reactive nanoarrays- based eNose	85 (a) 88 (b) 100 (c)	94 (a) 100 (b) 88 (c)	91 (a) 94 (b) 94 (c)
[66]	N = 182	OC	OC (n = 48), benign neoplasia (n = 86), healthy controls (n = 48)	Discrimination between: OC and other groups (a), OC and controls (b), neoplasia and controls (c), OC stages (d), early OC and controls (e)	Nanoscale NA-NOSE, coupled with GC-MS	71 (a) 79 (b) 72 (c)	71 (a) 100 (b) 64 (c)	71 (a) 89 (b) 69 (c) 72 (d) 75 (e)
[67]	N = 69	OC	OC (n = 51) of which: benign OCs (n = 18), malignant OCs (n = 33), controls (n = 18)	Discrimination between: benign vs. malignant tumors (a), malignant tumors vs. controls (b)	FAIMS	91.5 (a), 91.2 (b)	51.4 (a), 63.1 (b)	
[68]	N = 80	MM, ARD	MM (n = 20), ARD (n = 18), controls (n = 42)	To discriminate between patients and controls (a) and between MM, ARD and controls (b)	Cyranose 320	90 (a) 90 (b)	91 (a) 88 (b)	
[69]	N = 39	MM	MM (n = 13), professional asbestos exposure (n = 13), healthy subjects (n = 13)	To discriminate between MM and asbestos exposed (a) and between MM and controls (b)	Cyranose 320	92.3 (a)	85.7 (a)	80.8 (a) 84.6 (b)
[70]	N = 64	MM, ARD	MM (n = 14), benign ARD (n = 15), asymptomatic former AEx (n = 19), healthy controls (n = 16)	To discriminate between: MM and controls (a), MM and asbestos exposed without cancer (b), MM and benign ARD (c), MM and asbestos exposed without cancer + ARD (d), asbestos exposed without cancer and ARD (e)	Cyranose C320, Tor Vergata ENose, Common Invent Enose, Owlstone Lonestar	67 (a) 80 (b) 75 (c) 82 (d) 58 (e)	64 (a) 64 (b) 64 (c) 55 (d) 47 (e)	
[71]	N = 131	BlC, PC	BlC (n = 25), PC (n = 12), benign prostatic hypertrophy (n = 29), various urological conditions (n = 33)	Classification into the categories of cancer	ENQBE Enose, based on eight QCMs			100
[72]	N = 60	BlC	TCC (n = 30), controls (n = 30)	Discrimination between BlC and controls	Cyranose 320	93.3	86.7	
[73]	N = 41	PC	PC (n = 14), controls (n = 27)	Diagnostic concordance between eNose and prostate biopsy	eNose based on eight non-selective gas sensors, each coated with different metallo-porphyrins			85
[74]	N = 65	PC	PC (n = 50), benign prostatic hyperplasia (n = 15)	Discrimination between cancer and controls	MOS-based ChemPro® 100-eNose	78	67	
[75]	N = 41	PC	PC (n = 14), controls (n = 27)	Discrimination between: PC and controls	QMB-based eNose	N/A	93	N/A
[76]	N = 85	PC	PC (n = 32), controls (n = 53)	Discrimination between PC and controls	Aeonose	84	70	75
[77]	N = 36	BrC	Malignant BrC (n = 13), benign breast conditions (n = 16), healthy controls (n = 7)	Discrimination between the different groups	Nanoscale NA-NOSE			88.9
